# Genomic Dissection and Expression Profiling Revealed Functional Divergence in *Triticum aestivum* Leucine Rich Repeat Receptor Like Kinases (TaLRRKs)

**DOI:** 10.3389/fpls.2016.01374

**Published:** 2016-09-22

**Authors:** Shumayla, Shailesh Sharma, Rohit Kumar, Venugopal Mendu, Kashmir Singh, Santosh K. Upadhyay

**Affiliations:** ^1^Deparment of Botany, Panjab UniversityChandigarh, India; ^2^Deparment of Biotechnology, Panjab UniversityChandigarh, India; ^3^National Agri-Food Biotechnology InstituteMohali, India; ^4^Department of Plant and Soil Science, Fiber and Biopolymer Research Institute, Texas Tech UniversityLubbock, TX, USA

**Keywords:** duplication events, expression, neofunctionalization, phylogenetic groups, TaLRRKs, *Triticum aestivum*, stress

## Abstract

The leucine rich repeat receptor like kinases (LRRK) constitute the largest subfamily of receptor like kinases (RLK), which play critical roles in plant development and stress responses. Herein, we identified 531 *TaLRRK* genes in *Triticum aestivum* (bread wheat), which were distributed throughout the A, B, and D sub-genomes and chromosomes. These were clustered into 233 homologous groups, which were mostly located on either homeologous chromosomes from various sub-genomes or in proximity on the same chromosome. A total of 255 paralogous genes were predicted which depicted the role of duplication events in expansion of this gene family. Majority of TaLRRKs consisted of trans-membrane region and localized on plasma-membrane. The TaLRRKs were further categorized into eight phylogenetic groups with numerous subgroups on the basis of sequence homology. The gene and protein structure in terms of exon/intron ratio, domains, and motifs organization were found to be variably conserved across the different phylogenetic groups/subgroups, which indicated a potential divergence and neofunctionalization during evolution. High-throughput transcriptome data and quantitative real time PCR analyses in various developmental stages, and biotic and abiotic (heat, drought, and salt) stresses provided insight into modus operandi of TaLRRKs during these conditions. Distinct expression of majority of stress responsive *TaLRRKs* homologous genes suggested their specified role in a particular condition. These results provided a comprehensive analysis of various characteristic features including functional divergence, which may provide the way for future functional characterization of this important gene family in bread wheat.

## Introduction

The leucine rich repeat receptor like kinases (LRRKs) represent the largest subfamily of receptor like kinases (RLK), which are involved in diverse functions in plants (Shiu and Bleecker, 2001; Diévart and Clark, 2003; Shiu et al., 2004). Similar to the other RLK proteins, LRRKs also consisted of an extracellular domain (ECD) to perceive signals, a trans-membrane region to comfort the protein within the membrane, and a cytoplasmic protein kinase domain to stimulate the plant immunity via auto-phosphorylation, which is followed by phosphorylation of a specific substrate (Walker and Zhang, 1990; Shiu and Bleecker, 2001; Gou et al., 2010). The ECD of LRRKs consist of LRR domain, constituted by tandem repeats of 20–30 amino acid (AA) residues long leucine rich structural motifs (Jones and Jones, 1997; Matsushima and Miyashita, 2012). The LRR domain is stable among all proteins built from tandemly repeated motifs because of its hydrophobic solenoid inner core, where the conserved leucine and other aliphatic residues are present. This structure also favors the protein-protein interactions (Bella et al., 2008).

The LRRKs are involved in growth, development, and survival including organogenesis, morphogenesis, hormone signaling, and abiotic and biotic stress management in plants (Diévart and Clark, 2003; Li and Tax, 2013). For instance, BARELY ANY MERISTEM (BAM) 1–3 and CLAVATA1 are involved in apical growth, ERECTA-LIKE (ERL) 1–2 in organ growth, plant architecture, and stomatal and floral development, BRI1 in vascular development, HSL in flower abscission, FEI2 in cell wall biosynthesis, and GSO and RPK1 in embryogenesis, (Clark et al., 1997; Delgado et al., 2004; Shpak et al., 2004; Deyoung et al., 2006; Nodine et al., 2007; Stenvik et al., 2008; Tsuwamoto et al., 2008; Xu et al., 2008). The LRRK FLS2 and EFR regulate the plant immunity and act as receptor for flagellin and EF-Tu for plant defense (Gomez and Boller, 2000; Zipfel et al., 2006). PSKR1, BAK1, BRI1, and SERK of arabidopsis are also reported to be involved in biotic stress and defense responses (Nam and Li, 2002; Roux et al., 2011; Wang et al., 2011; Mosher et al., 2013; Wu et al., 2016). The differential expression of LRRK genes during abiotic stresses in various plants like arabidopsis, rice, and potato has been reported (Diévart and Clark, 2004; Osakabe et al., 2005; Park et al., 2014; Wu et al., 2016). The GHR1 of arabidopsis is induced by ABA and H2O2, while RPK1 by drought, salt, abscisic acid (ABA), and low temperature (Osakabe et al., 2005; Hua et al., 2012). OsGIRL1 is involved in salt, osmotic, heat, and gamma radiation stress in *Oriza sativa* (Park et al., 2014). Several other differentially expressed LRRKs are known in response to various biotic and abiotic stresses in plants, whose specific functions need to be established in future studies. Moreover, some LRRKs like SOMATIC EMBRYOGENESIS RECEPTOR-LIKE KINASE (SERK) and ERECTA of arabidopsis (Godiard et al., 2003; Shpak et al., 2004; Colcombet et al., 2005) possess dual functions, which are due to either recognition of numerous ligands by a single receptor or cross talk between various pathways (Torii et al., 1996; Afzal et al., 2008). The LRRKs are mostly evolved by extensive gene/domain duplication; therefore they have probably acquired diverse functions through neofunctionalization (Jones and Jones, 1997; Walsh, 2003).

In plants, the functional characterization of *LRRK* genes are largely performed in model plant arabidopsis (Shiu and Bleecker, 2001; Shiu et al., 2004; Wu et al., 2016). In recent years, the study has been certainly extended, but it is still limited to a few plants like rice, tomato brassica, poplar, and soybean (Sun and Wang, 2011; Zan et al., 2013; Rameneni et al., 2015; Wei et al., 2015; Zhou et al., 2016). It is probably due to lack of genomics information, large number of genes and complication in applicability of functional genomics tools in various other plants. The *Triticum aestivum* (bread wheat) is one of the main food crops of the world, which is being consumed by about one-third population as a major source of calorie. It is used in the preparation of bread, biscuit, cake, pastries, and several other food materials. The *T. aestivum* comprises a composite allohexaploid genome (2n = 6x = 42; AABBDD), which was originated by hybridizations of three diploid A, B, and D sub-genomes (Marcussen et al., 2014). The characterization of *LRRK* gene family has not been performed in *T. aestivum*, despite being a staple food crop. The International Wheat Genome Sequencing Consortium (IWGSC) has reported the chromosome based genome information of *T. aestivum* in 2014 (IWGSC, 2014). Further, numerous developmental stage specific and stress related high-throughput RNA sequence (RNA seq) data of *T. aestivum* have been reported in recent years (Zhang et al., 2014, 2016; Liu et al., 2015; Pingault et al., 2015). The availability of genomic information and these RNA seq data enabled us for the identification and characterization of *LRRK* gene family in *T. aestivum*. Since the *LRRK* genes play vital roles in development and stress tolerance in plants (Shiu et al., 2004), their characterization will be valuable in future genetic improvement program of *T. aestivum*.

Herein, the entire *TaLRRK* gene family was identified using the genomic data of *T. aestivum* (IWGSC, 2014) and comprehensive analyses were performed. The gene family was characterized for genome wide distribution, occurrence of duplication events, homologous, and orthologous genes identification, annotation, and gene ontology (GO) mapping, gene, and protein structure, domain, and motif organization, and evolutionary relationships. The expression profiling during various developmental stages and stress conditions was performed to understand their role in these biological processes. To the best of our knowledge, this is the first report on genome wide characterization of *LRRK* genes in *T. aestivum*.

## Materials and methods

### Identification, annotation, and chromosomal distribution

To identify the entire TaLRRKs of *T. aestivum*, following two approaches were used. First, we performed the blastp search of known LRRK protein sequences of arabidopsis and rice (Shiu and Bleecker, 2001; Shiu et al., 2004; Sun and Wang, 2011) against high confidence protein model sequences of *T. aestivum* obtained from the IWGSC (http://www.wheatgenome.org/, http://wheat-urgi.versailles.inra.fr/Seq-Repository/Genes-annotations) (IWGSC, 2014). Second Hidden Markov model (HMM) scan was performed using LRR (LRR_8, PF13855; LRR_NT II, PF08263; LRR_4, PF012799; LRR_6, PF13516; LRR_9, PF14580; LRR_I, PF00560; LRR_V, PF13306), and protein kinase (PF00069) pfam profiles obtained from pfam database (ftp://ftp.sanger.ac.uk/pub/databases/Pfam) (Finn et al., 2014). The unique set of sequences having both LRR and protein kinase domain were considered as putative TaLRRKs. These sequences were further validated by blast search against NCBI-conserved domain database (http://www.ncbi.nlm.nih.gov/Structure/bwrpsb/bwrpsb.cgi). Further, the domain organization was confirmed using SMART (http://smart.embl-heidelberg.de/) and Prosite-Scan (http://prosite.expasy.org/scanprosite/) servers. The annotation of identified sequences was performed by blast search (*e*-value 10^−6^) against the NCBI non-redundant protein, UNIPROT/SWISSPROT, and UNIPROT-UNIREF databases. The BLAST2GO tool was used for the gene ontology mapping (Conesa and Gotz, 2008). The chromosomal location was obtained by blastn search of *TaLRRK* gene sequences against chromosome sequences of *T. aestivum* available at Ensembl Plants (http://plants.ensembl.org/Triticum_aestivum/Info/Index) and URGI (http://urgi.versailles.inra.fr/blast/) servers. The sub-genomic (A, B, and D) categorization was based on gene model sequence identifier. MapInspect (http://mapinspect.software.informer.com/) was used to prepare the chromosome maps.

### Prediction of homologs, orthologs, duplication events, and phylogenetic analysis

The homologous sequences were predicted on the basis of two criteria- (i) bi-directional blastn search (*e*-value 10^−10^) with ≥90% sequence similarity between *TaLRRK* gene sequences and (ii) clustering with the same *T. aestivum* unigene sequence (http://www.ncbi.nlm.nih.gov/UniGene). The sequences following both the criteria were considered as homologs. A non-redundant nomenclature was also performed as per the clustering and homology between sequences. The orthologous genes were predicted using best bidirectional blast hit (*e*-value 10^−10^) approach. The *TaLRRK* gene sequences were used for blastn search against the *LRRK* sequences of rice and arabidopsis. The intra-chromosomal duplication events were predicted by blastn search (*e*-value 10^−10^) with ≥80% sequence similarity. The genes located within 5 Mb region on the same pseudo-molecule (chromosome) were considered as tandemly duplicated (TD), while those located beyond 5 Mb were designated as segmentally duplicated (SD) (Shumayla et al., 2016). The evolutionary relationship was analyzed by neighbor joining method with 1000 bootstrap replicates using MEGA7 (Kumar et al., 2016).

### *In-silico* characterization

The exon/intron configuration was obtained by aligning coding sequences (CDS) with genomic sequence of each gene obtained from Ensemble plants (http://plants.ensembl.org/Triticum_aestivum/Info/Index). The conserved domains were predicted by blast search at NCBI conserved domain database (http://www.ncbi.nlm.nih.gov/Structure/bwrpsb/bwrpsb.cgi) (Marchler et al., 2015). Motifs were predicted by multiple sequence alignment subjected to the Web Logo (http://weblogo.berkeley.edu/logo.cgi) and consurf blast (http://consurf.tau.ac.il) server. The molecular weight, pI, signal peptide, cellular localization, and transmembrane region were predicted using Expasy MW/pI (http://web.expasy.org/compute_pi/), SignalP 4.1 (http://www.cbs.dtu.dk/services/SignalP/), CELLO v.2.5 (http://cello.life.nctu.edu.tw/), and TMHMM v2.0 (http://www.cbs.dtu.dk/services/TMHMM/) servers, respectively (Yu et al., 2004, 2006). MAFFT, (http://www.ebi.ac.uk/Tools/msa/mafft/), muscle (Edgar, 2004) and ClustalW (http://www.ebi.ac.uk/Tools/msa/clustalw2/) were used for the multiple sequence alignments. The venn diagram was made using Venny (Oliveros, 2007-2015).

### Genome wide expression analysis

The tissue specific expression profiling of *TaLRRKs* was performed using high throughput RNA-seq data (https://urgi.versailles.inra.fr/files/RNASeqWheat/) generated in two biological replicates from root, stem, leaf, spike, and grain, each with three developmental stages (accession number ERP004714) (Pingault et al., 2015). The effect of biotic stress of fungus *Blumeria graminis f*. sp. *tritici* (Bgt) and *Puccinia striiformis* f. sp. *tritici* (Pst) on the expression of *TaLRRKs* in leaves was studied using RNA seq data (accession number PRJNA243835) developed after 24 h of inoculation in triplicates (Zhang et al., 2014). Expression analysis under heat, drought and their combination was performed using RNA seq data (accession number SRP045409) generated from leaves after 1 and 6 h of incubation in two biological replicates (Liu et al., 2015). The effect of salt (NaCl) stress was analyzed in root tissue using RNA seq data (accession number SRP062745) produced in three biological replicates after 6, 12, 24, and 48 h of treatments (Zhang et al., 2016). The quality filtered RNA seq reads were mapped to *TaLRRK* gene sequences with 100% query coverage and sequence similarity. The abundance of mapped reads to each gene was counted using locally made python script and used to calculate RPKM (reads per kilobase per million mapped reads) value as described (Mortazavi et al., 2008; Upadhyay et al., 2015; Shumayla et al., 2016). The heat-maps showing expression profiles were generated using Hierarchical Clustering Explorer 3.5 (http://www.cs.umd.edu/hcil/hce/).

### Validation of gene expression by qRT-PCR

The organ specific expression of selected *TaLRRK* genes were validated in root, shoot, leaf, and seeds (from two developmental stages, Z_71 and Z_75; Z denotes Zadoks scale) of *T. aestivum* cv. Chinese Spring following the method described (Shumayla et al., 2016). The heat, drought, and their combination stress responsive expression was validated in leaves subjected to 1 and 6 h of incubation, while the effect of salt (NaCl) stress on selected *TaLRRK* genes expression in root tissue was analyzed after 6, 12, 24, and 48 h of treatments, as described (Liu et al., 2015; Zhang et al., 2016). The seeds were surface sterilized using 1% sodium hypochlorite solution, washed several times using autoclaved Milli-Q water and placed on moistened filter paper in petri dishes for 2 days at 20°C. The uniformly grown seedlings were transferred to half strength Hoagland solution in hydroponic condition and grown in growth chamber under 22/18°C (day/night), 16/8 h (light/dark), and 50% relative humidity. The seedlings were subjected to various stress conditions after 1 week. The drought stress was applied using 20% PEG solution in place of water (Dhanda et al., 2004; Soltani et al., 2006), while heat stress was applied by incubation of plants at 40°C in growth chamber. The leaves samples were collected separately after 1 and 6 h of incubation under heat (40°C), drought (20% PEG), and combination (40°C and 20% PEG) stress, frozen in liquid nitrogen, and stored at −80°C. The salt stress was applied by adding 150 mM NaCl to the hydroponic solution and root samples were collected after different hours of treatment. The normal grown seedlings were used as control and all the experiments were performed in three biological replicates.

The total RNA was isolated from normal and stress treated samples using the RNeasy Plant Mini Kit (Qiagen, USA) and DNA contaminations were removed using RNase free kit (Ambion USA). Superscript III First Strand cDNA Synthesis Kit (Invitrogen, USA) was used for the synthesis of cDNA and quantitative real time (RT) PCR was performed with gene specific primers (Table S1) using ABI7500 Fast System (Applied Biosystems, USA) following the method described in our earlier studies (Upadhyay et al., 2013, 2016; Shumayla et al., 2016). The amplification of 18S_rRNA gene (accession number AJ272181.1) was used as an internal control (Bhati et al., 2014; Shumayla et al., 2016) and transcripts abundance was calculated using ^▴▴^Ct method (Livak and Schmittgen, 2001).

## Results

### Isolation and chromosomal localization of *TaLRRK* genes

The blast search of LRRK protein sequences of arabidopsis and rice, HMM blast and pfam blast was used to comprehensively identify the LRRK sequences from *T. aestivum*. A total of 531 non-redundant *TaLRRK* genes were identified after an extensive blast search. The presence of an intracellular kinase (pfam00069) domain and an extracellular LRR domain in each TaLRRKs was confirmed by blast search against pfam and NCBI-conserved domain databases. These were further confirmed by PROSITE-Scan and SMART server.

**Figure 1:**
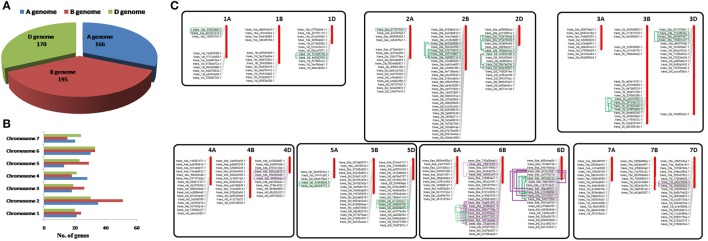
**Distribution of *TaLRRK* genes on A,B,D sub-genomes and chromosomes, and duplication events**. Figure shows the number of *TaLRRK* genes located on A, B, and D sub genomes **(A)** and various chromosomes **(B)**. The physical localization of *TaLRRK* genes on various chromosomes **(C)** is performed for the genes located on reported pseudo-molecules (chromosome) sequences, whereas those occurred on scaffolds are not mapped. The predicted intra-chromosomal duplication events are highlighted and connected by boxes and lines. The purple and green colors indicate segmental (SD) and tandem duplication (TD), respectively.

The allohexaploid genome (2n = 6x = 42; AABBDD) of *T. aestivum* was originated by hybridization events of three diploid A, B, and D sub-genomes (Marcussen et al., 2014). Genome wide distribution indicated the sharing of 166, 195, and 170 *TaLRRK* genes from A, B, and D sub-genomes, respectively (Figure 1A; File S1). Since the *T. urartu* and *Aegilops tauschii* are reported as donor of A and D sub-genome, (Marcussen et al., 2014) and their genome sequences are recently reported (Jia et al., 2013; Ling et al., 2013), the number of *LRRK* genes in these plants was also analyzed. A total of 217 and 248 *LRRK* genes were predicted in *T. urartu* and *Ae. tauschii*, respectively (File S2).

The chromosomal distribution on the basis of gene model identifier indicated the occurrence of *TaLRRK* genes on each chromosome but at different frequencies (Figure 1B; File S1). A maximum of 51 genes were located on chromosome 2B, while a minimum 13 genes on chromosome 5A. The physical localization of *TaLRRK* genes was analyzed by blastn search against each chromosome sequence. Out of 531 *TaLRRK* genes, 356 were located on pseudo-molecules (chromosomes), however remaining 175 were mapped on scaffolds (Figure 1C; File S3). This might be due to the unavailability of complete genome and chromosome sequence of *T. aestivum*.

### Prediction of homologs, paralogs, and orthologs

On the basis of high similarity with each other and, grouping with a common unigene cluster, the *TaLRRK* genes were clustered into 233 distinct groups with one or more sequences. These clustered sequences were considered as putative homologs on the basis of their high similarity with each other. The clustering information was also used for the nomenclature of these genes (File S3). Most of the clusters consisted of at least one sequence from the homeologous chromosomes of A, B, and D sub-genomes, which might also be considered as homeologous genes. The largest cluster (LRRK70) consisted of 20 homologous sequences, while 99 *TaLRRK* sequences were distinct without any homolog.

A total of 255 duplication events (DEs) were predicted in *TaLRRKs*, out of them only 66 could classified as TD and SD, due to the physical localization of paralogous gene pairs on pseudo-molecules (Figure 1C; File S4). However, 189 DEs could not be categorized as TD or SD, because one or both the genes from paralogous pair were located on scaffold. A total of 50, 128, and 77 DEs were predicted on A, B and D sub-genomes, respectively. These DEs were localized on all the chromosomes except chromosome 7A. A maximum of 54 DEs occurred on chromosome 1B, while only one DE was located on chromosome 4B.

The reported *LRRK* gene sequences of rice and arabidopsis was downloaded from Rice Genome Annotation Project (rice.plantbiology.msu.edu/) and The Arabidopsis Information Resource (TAIR) (http://www.arabidopsis.org) databases and used for the prediction orthologous sequences of *TaLRRK* genes by the best bidirectional blast hit approach as described earlier (Herve et al., 1999; Shumayla et al., 2016). A total of 104 and 176 nearest orthologous genes of *TaLRRKs* were predicted from arabidopsis and rice, respectively (File S5). The functional correlation of orthologous gene pairs has been discussed in later section.

### Annotation and gene ontology analysis

**Figure 2:**
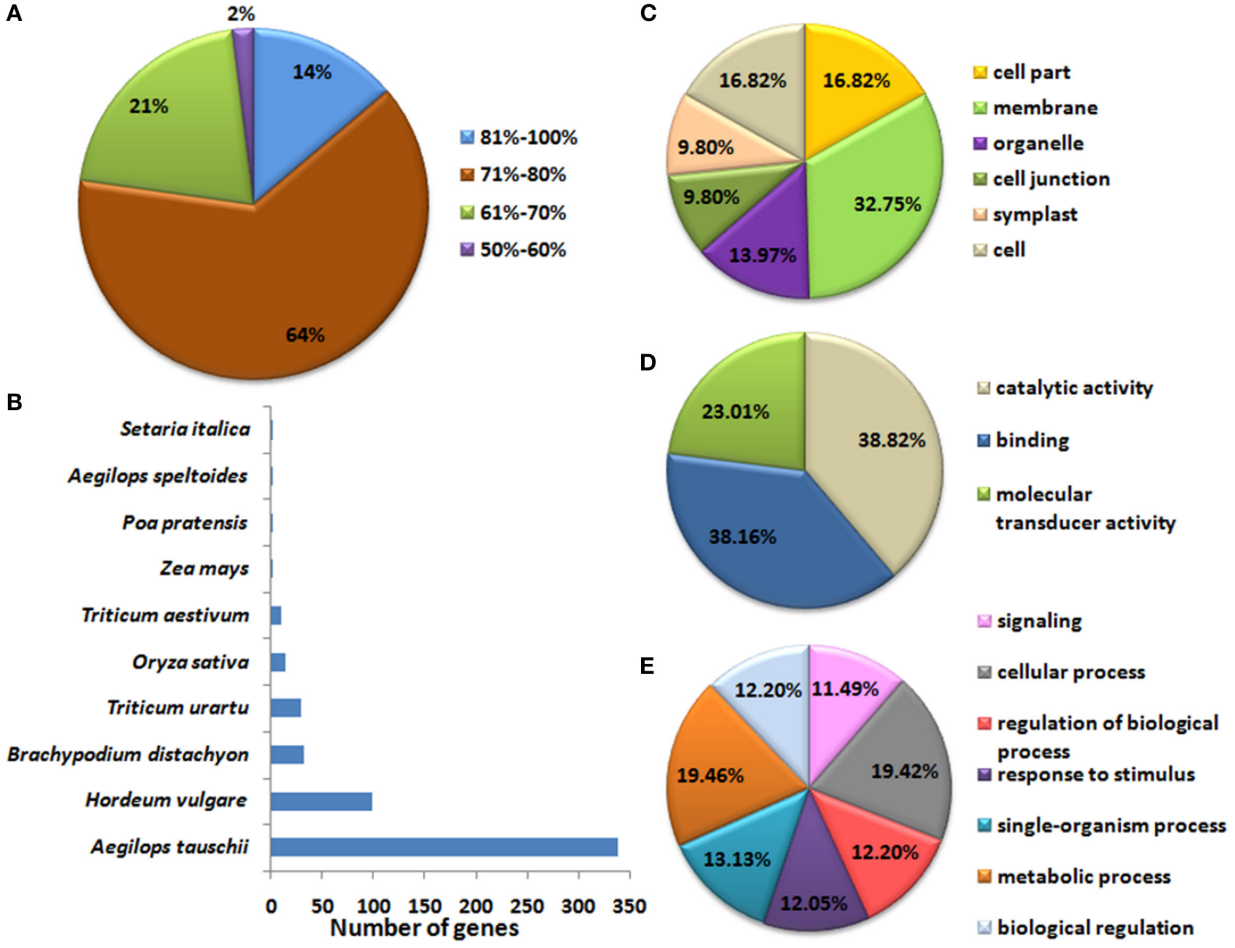
**Blast2Go annotation statistics and gene ontology (GO) mapping of *TaLRRK* genes**. Figure shows **(A)** percent similarity of *TaLRRK* genes with the known sequences in the NCBI-nr database during annotation, and **(B)** top blast hit species. GO mapping shows functional categorization of TaLRRKs into **(C)** cellular components, **(D)** molecular functions and **(E)** biological processes.

The identified *TaLRRK* genes were further validated by their functional annotation and gene ontology (GO) mapping. Annotation was performed by blast search against various databases (Files S6, S7). About 98% *TaLRRKs* were annotated with ≥60% similarity against NCBI non-redundant protein database (Figure 2A). The top blast hit plant species were *Ae. tauschii* (339) and *Hordeum vulgare* (99) (Figure 2B). The blast search of *TaLRRKs* showed homology with 336 and 89 distinct protein sequences from UNIREF_100 and UNIPROT databases, respectively (File S7). Most of the *TaLRRKs* were annotated as leucine rich repeat receptor like serine threonine protein kinase. Some of them were annotated as homologs of arabidopsis BRASSINOSTEROID INSENSITIVE 1 (BRI1), CLAVATA1, ERECTA LIKE 1 (ERL1), ERL2, RECEPTOR LIKE PROTEIN KINASE2 (RPK2), GASSHO1 (GSO1), TMK1, MRH1, PHYTOSULFOKINE RECEPTOR 1-LIKE (PSKR1), SOMATIC EMBRYOGENESIS RECEPTOR KINASE (SERK1), and other proteins.

The GO mapping was performed using BLAST2GO tool (Conesa and Gotz, 2008). A total of 6709 GO terms were mapped to TaLRRK proteins, which were classified into cellular components (2662), biological processes (2687), and molecular functions (1360) categories. The GO term “membrane” was obtained for almost each TaLRRK in cellular component category (Figure 2C). In molecular function, binding (38%), and catalytic activity (38%) was enriched category (Figure 2D). In biological processes, GO terms were evenly distributed in various categories like signaling, response to stimulus, cellular, and metabolic processes (Figure 2E), which depicted diverse role of TaLRRKs in plant development and defense mechanism. Similar GO categorization is also reported for brassica LRRKs (Rameneni et al., 2015).

### Gene and protein characterization

**Figure 3:**
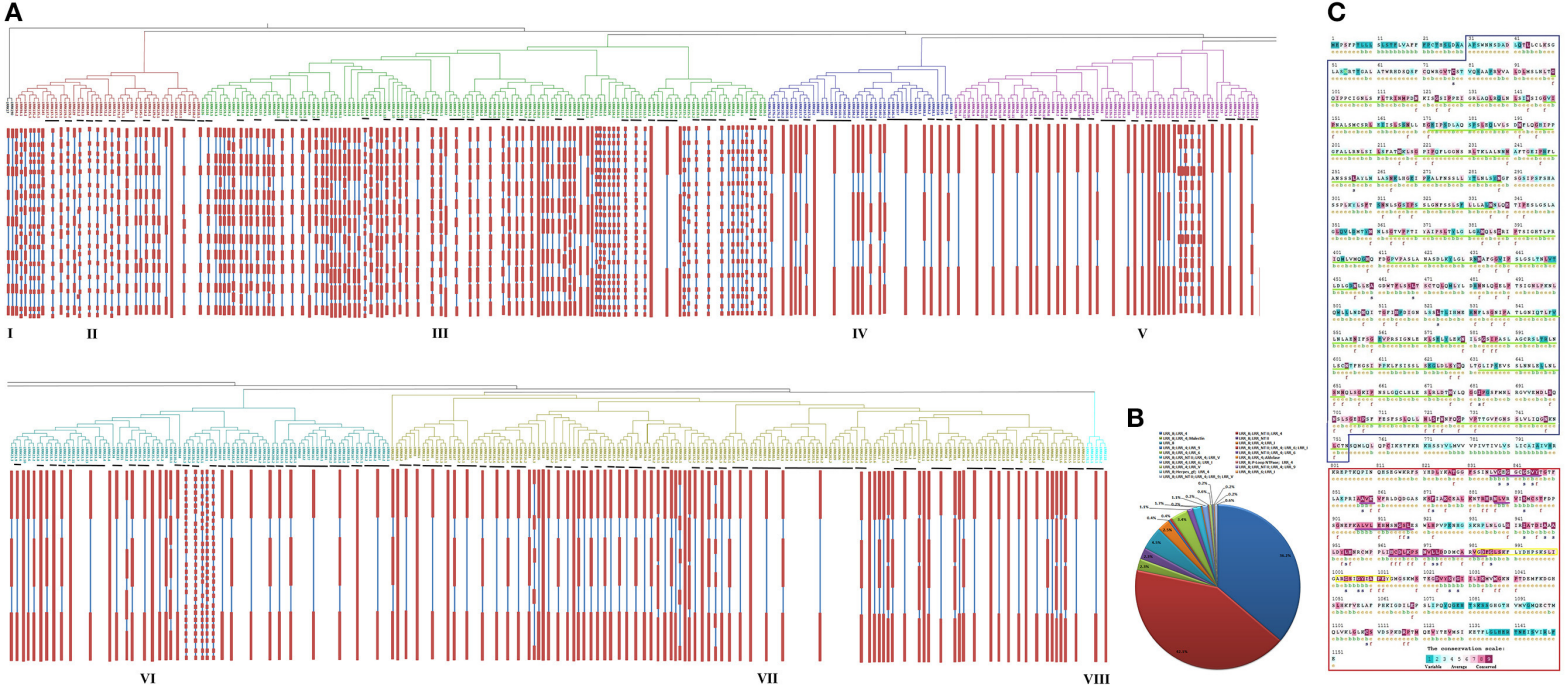
**Gene and protein structure analysis. (A)** Exon/intron distribution in *TaLRRK* genes grouped under various classes of phylogeny is shown. Red and Blue colors indicate the occurrence of exon and intron in various genes. **(B)** Extracellular domain composition of TaLRRK proteins obtained by blast search at pfam and NCBI conserved domain databases. **(C)** Conservation of various domain and motifs in TaLRRK proteins obtained by multiple sequence alignment subjected to ConSurf blast. The extracellular LRR domain and Kinase domain are shown in blue and red color boxes, respectively. The LRR motifs are underlined with green color in LRR domain. In kinase domain, ATP binding site/active sites are underlined by purple color, while activation loop is shown in yellow boxes.

The gene structure was analyzed in terms of exon/intron organization. The number of exons in *TaLRRK* genes varied from one to 28 (File S3). Out of 531 *TaLRRK* genes, 71 (13%) were intron less, and 198 (37%) consisted of single intron (Figure 3A). A maximum of 27 introns were detected in *LRRK69.1*, followed by *LRRK136.1*, and *LRRK193.2* with 26 introns each.

The LRRK186.1 (1315 AA) and LRRK 51.2 (169 AA) were the longest and the smallest TaLRRK proteins. However, the average coding sequence and protein length was 2386 bp and 794 AA residues, respectively. The average molecular weight of TaLRRKs was found to be ~80 kDa. The isoelectric point range of TaLRRKs was 4.8–9.9; where LRRK227 had the highest pI (File S3). Out of 531 TaLRRKs, 510 were consisted of one or more transmembrane regions. A maximum of three transmembrane regions were found in six TaLRRKs, while single transmembrane was detected in 401 TaLRRK proteins. Analysis at SignalP (http://www.cbs.dtu.dk/services/SignalP/) server indicated the presence of signal peptide in 244 TaLRRK proteins. Most of the TaLRRK proteins were predicted to be localized in plasma membrane (324), which was followed by extracellular (93), and nuclear (65) localization. A few were localized in chroloplast (29), cytoplasm (20), and mitochondria (5) (File S3).

To explore the diversification and functional potentials of TaLRRKs, their domains and motifs were analyzed. In total, 12 domains were predicted including kinase domain, which were present in various combinations (File S8). The LRR_8 and kinase domains were found in each TaLRRK proteins, which were followed by LRR_4 (494) and LRR_NT II (258). Besides these major domains, some TaLRRK also consisted of LRR_6 (34), LRR_1 (22), LRR_5 (15), LRR_9 (6), Malectin (12), P-loop NTPase (1), Aldolase II (1), and Herpes_gE (1) domains. The majority (~42%) of TaLRRKs consisted of the combination of LRR_8; LRR_NT II; LRR_4 domains, followed by LRR_8; LRR_4 domains (~36%) along with the kinase domain (Figure 3B).

The TaLRRKs were further analyzed for conserved nature of major domains and various other motifs by multiple sequence alignment subjected to the Web Logo software and consurf blast. The kinase domain was found highly conserved in comparison to the LRR domain (Figure 3C). Several conserved motifs were detected in both LRR and Kinase domains of TaLRRK (Figure 3C; Table S2). Numerous LRR motifs were found in TaLRRKs, most of them consisted of similar organization of conserved amino acids (GxIPxxLxxLxxLxxLxLxxN). The highly conserved amino acids positions were G at 1st, P at 4th, L at 7th, 10th, 13th, 16th, and 18th, and N at 21st. A few conserved motifs were also predicted in kinase domain, which were localized around the ATP binding sites, active sites, substrate binding sites, and activation loop (Figure 3C).

### Phylogenetic analysis

**Figure 4:**
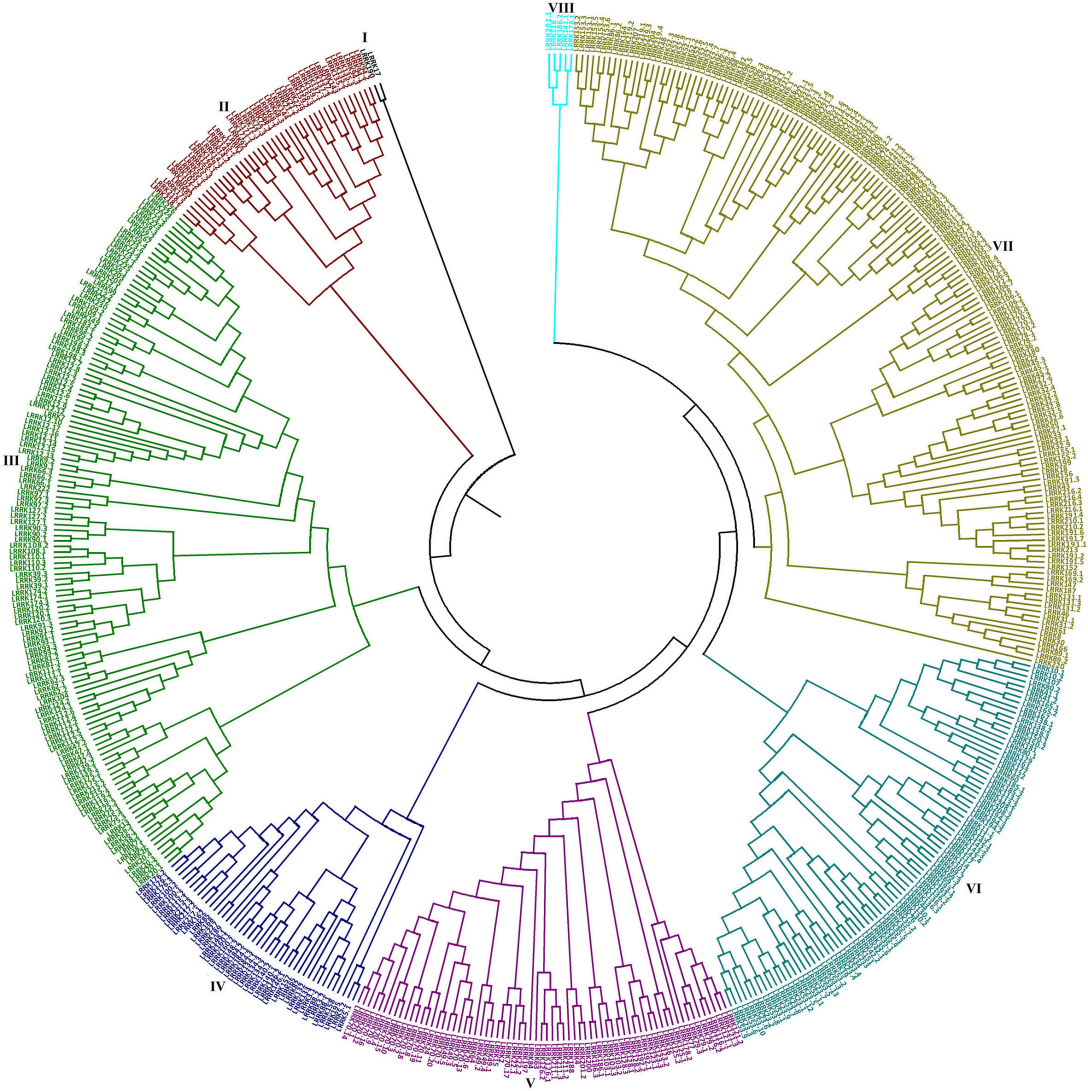
**Phylogenetic analysis of TaLRRKs**. The evolutionary relationship between TaLRRK proteins was analyzed by neighbor joining method using MEGA 7.0. Figure shows classification of TaLRRK proteins into eight major groups, which are shown with different colors.

The evolutionary relationship between 531 TaLRRK proteins was studied by constructing a phylogenetic tree using MEGA7 (Kumar et al., 2016). The TaLRRKs were classified into eight main groups, which were further divided into subgroups and clades (Figure 4). The size of each group varied significantly from two members in group I to 157 in group 7. The predicted homologous TaLRRK sequences were grouped together with high bootstrap value in phylogenetic tree, which also supported the proposed nomenclature pattern. Majority of subgroups or clades comprised either predicted homologous sequences located on homeologous chromosome (A, B, and/or D sub-genome), or predicted paralogous sequences probably originated by duplication events and located on same chromosome. For instance, LRRK12 with 17 homologs was clustered in a clade of group III, while LRRK70 with 20 homologs was grouped together in group V.

The phylogenetic groups were also analyzed in terms of exon/intron pattern (Figure 3A) and various other features (Table S3). Though the range of number of introns in each group was overlapping, the closely clustered TaLRRKs in various subgroups and clades showed a similar exon/intron organization pattern. The analysis of various other characteristic features indicated variation in average molecular weight, pI and signal peptide between different groups, but the range was overlapping (Table S3). The domain architecture and LRR repeats were also found consistent in closely clustered sequences in each group (File S9).

### Expression analysis in various organs and developmental stages

**Figure 5:**
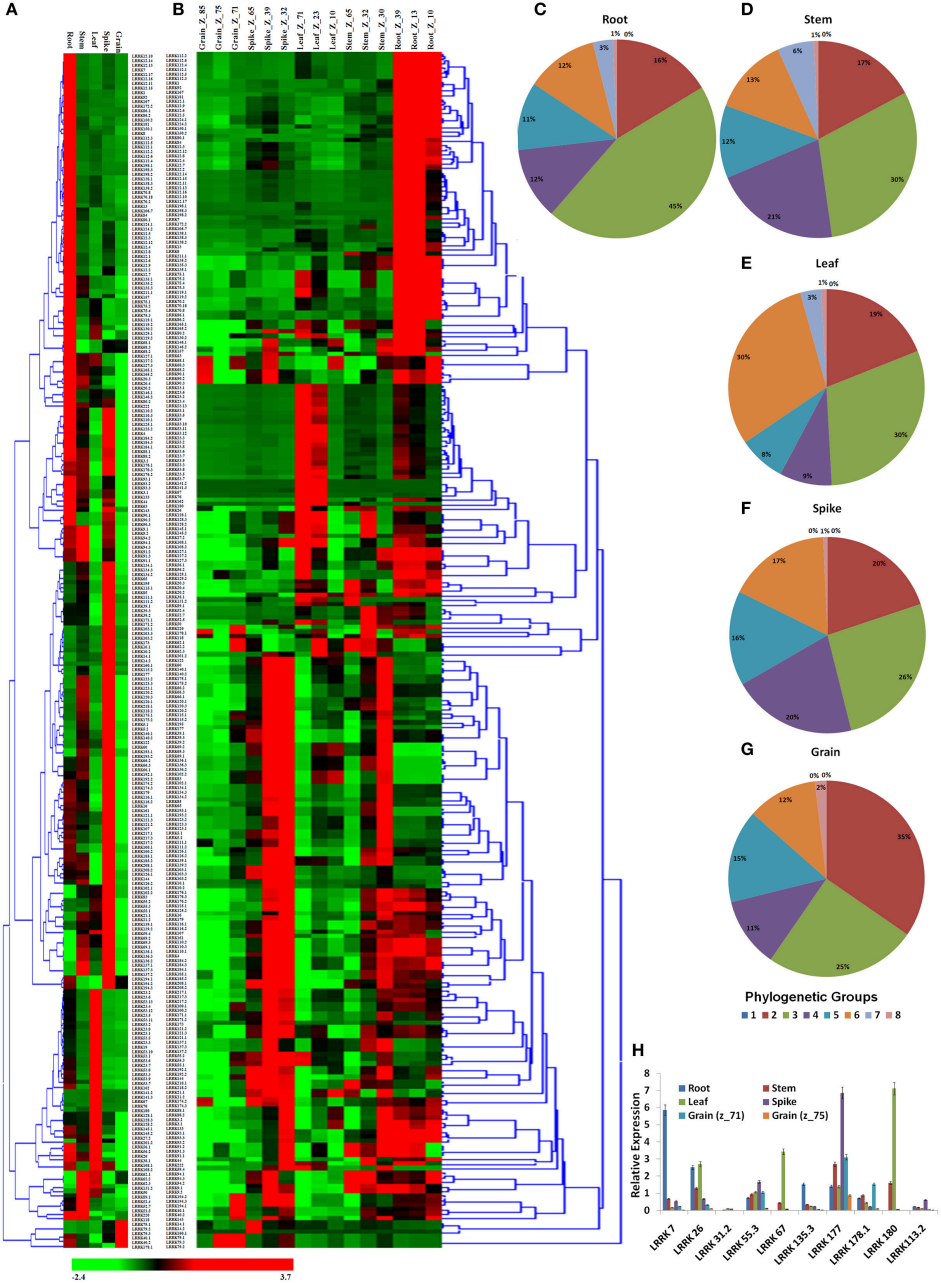
**Relative expression profile of *TaLRRK* genes in various organs and their three developmental stages**. Heat map shows relative expression profile of *TaLRRKs* in five different organs **(A)** and three developmental stages of each organ **(B)**. Phylogenetic classification of genes with expression value ≥10 RPKM in root **(C)**, stem **(D)**, leaf **(E)**, spike **(F)**, and grain **(G)** shows evolutionary relationship in genes expressing in same organ. Quantitative real time PCR analysis of 10 selected *TaLRRK* genes **(H)** shows relatively similar expression pattern, as observed with transcriptome data.

The expression profiling of *TaLRRK* genes in various organs and their developmental stages was performed using high throughput RNA-seq data (Pingault et al., 2015). A total of 263 genes were used for expression analysis, because their expression values were ≥10 RPKM in one or more developmental stages (File S10). The organ wise expression profiling indicated that majority of *TaLRRK* genes were highly expressed in root (~40) and spike (~35%) compared to the other organs (Figure 5A). Only six genes (three homologs of *LRRK79*, two of *LRRK40*, and *LRRK178.1*) showed higher expression in grain than in other organs. The real time (RT) PCR validation of certain genes like showed a similar relative expression pattern (Figure 5H). For instance, the genes *LRRK7, LRRK177*, and *LRRK180* were relatively highly expressed in root, spike and leaf, respectively. However, *LRRK26* showed similar expression in root and leaf. The phylogenetic grouping of highly expressed genes from various organs indicated that majority of them belong to group III in root (45), stem (30), and spike (26%). In leaf, high expressing genes were equally (30%) distributed in group III and VI, while majority of them (35%) belongs to group II in grain (Figures 5C–G).

The developmental stage specific expression profiling revealed that the highly abundant genes in root were mostly belonged to Z_13 and Z_39 stages; while in stem, leaf and spike from Z_30, Z_71 and Z_32 stages, respectively. In grain, *LRRK79* was exclusively highly expressed in Z_71 and Z_75 stages, while *LRRK68* and *LRRK90* were highly expressed in Z_85 (Figure 5B). Other stage specific highly expressed genes were *LRRK94.3* (root and stem), *LRRK180* (Z_23 and Z_71 stage of leaf) and *LRRK193.1* (Z_32 stage of spike). The detail expression value of each gene in developmental stage specific manner is listed in File S10.

### Gene expression analysis under biotic stress

**Figure 6:**
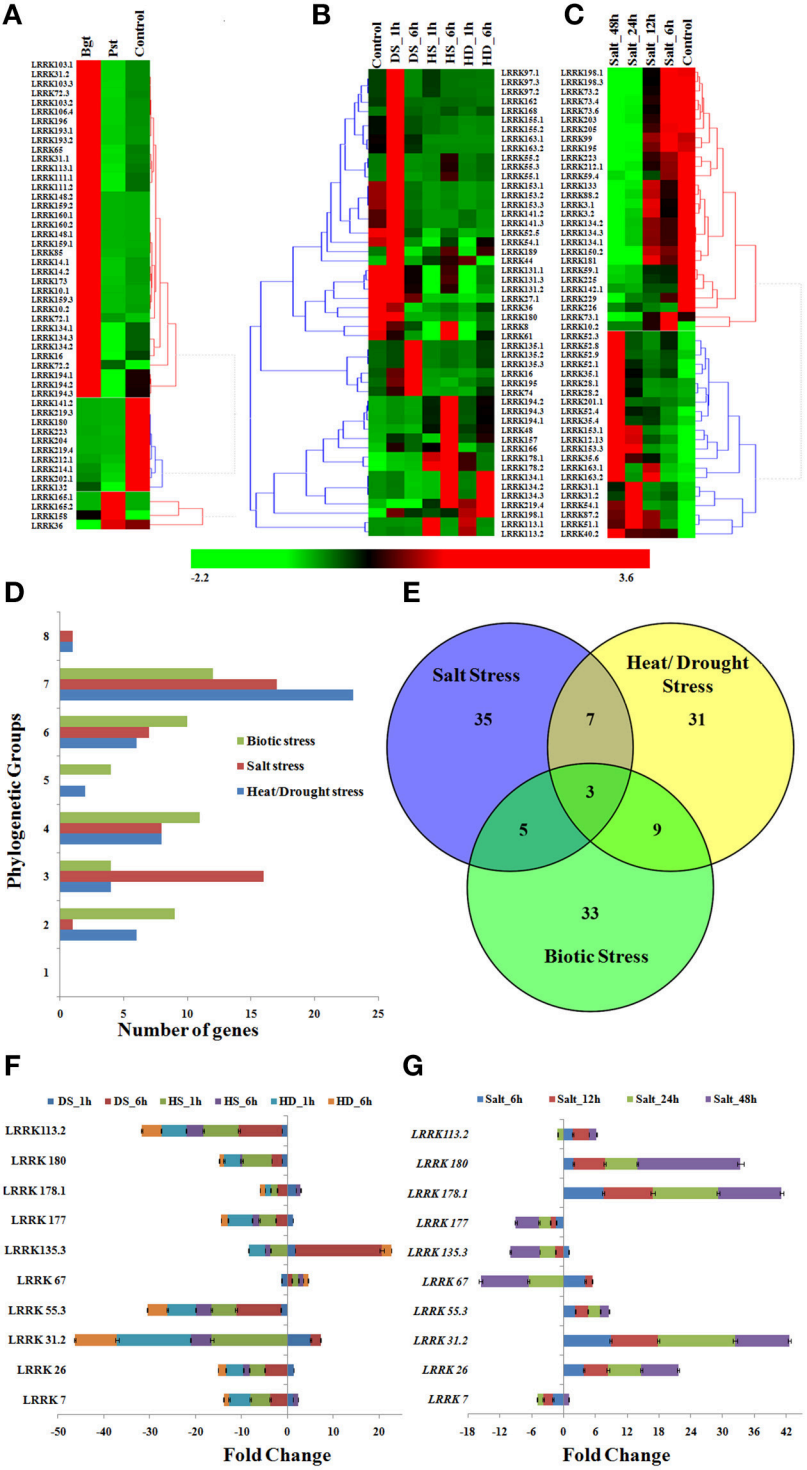
**Expression profile of *TaLRRK* genes under biotic and abiotic stresses**. The heat map shows relative expression of top 50 affected genes under **(A)** biotic stress, **(B)** heat/drought stress, and **(C)** salt stress. The symbols are as follows- Bgt; after *Blumeria graminis* infection, Pst; after *Puccinia striiformis* inoculation, HS; heat stress, DS; drought stress, HD; combination of heat and drought stress. **(D)** The phylogenetic classification of top affected genes under biotic and abiotic stresses. **(E)** The venn diagram shows the number of exclusive and common top affected genes under various biotic and abiotic stresses. Quantitative real time PCR analysis of 10 selected genes under heat/drought **(F)** and salt **(G)** stress shows similar expression pattern as detected from transcriptome data analysis.

A total of 450 *TaLRRK* genes were found as differentially expressed (≥2 folds change) after infection of fungal pathogens, *Blumeria graminis* f. sp. *tritici* (Bgt) and *Puccinia striiformis* f. sp. *tritici* (Pst), which causes powdery mildew and stripe rust diseases, respectively (File S11). The top 50 differentially expressed genes were used for further analysis. It was observed that most of the affected genes were up-regulated in Bgt infection, while down-regulated in Pst infection (Figure 6A). A few highly affected genes were- *LRRK158, 165.1*, and *165.2* (8 fold up), and *LRRK202.1* (225 fold down)*, 134.1-3* (166 fold down) in Pst infection; *LRRK148.1* (287 fold up) and *159.2* (183 fold up), and *LRRK204* (179 fold down) and *219.4* (140 fold down) in Bgt infection. Further, we observed that ~50% of highly up-regulated genes were exceptionally low expressing during normal uninfected conditions. For instance, the *LRRK158, 160.1*, and *160.2* showed extremely low expression during normal condition, while these genes were more than 100 fold up-regulated after fungal infection, which indicated their specific role in these stress management.

### Gene expression analysis under abiotic stress

We studied the expression of *TaLRRK* genes under heat (HS), drought (DS), and their combined stress (HD) conditions. A total of 492 genes showed ≥2 folds (F) up or down regulation during HS, DS, and HD (File S12), top 50 differentially expressed genes from them were analyzed in detail. Majority of the affected genes (~50%) were up-regulated in DS after 1 h, which became either normalized or down-regulated after DS 6 h (Figure 6B). However, in case of HS, most of the down-regulated genes such as *LRRK8, 61, 134*, and *157*, and slightly up-regulated genes like *LRRK48, 166*, and *196* were highly up-regulated in HS 6 h. In case of HD, assorted expression of *LRRK* genes was observed as compared to the DS and HS. The highly up (↑) and down (↓) regulated genes in various stress were- *LRRK168* (21 F↑) and *LRRK36* (2 F↓) in DS after 1 h, *LRRK135.3* (18 F↑*)* and *LRRK36* (85 F↓) in DS after 6 h, *LRRK113.1* (30 F↑) and *LRRK 141.2* (88 F↓) in HS after 1 h, *LRRK194.2* (44 F↑), and *LRRK141.3* (137 F↓) in HS after 6 h, *LRRK113.1* (15 F↑) and *LRRK180* (146 F↓) in HD after 1 h, and *LRRK194.2* (11 F↑) and *LRRK141.3* (94 F↓) in HD after 6 h, respectively. Further, about half of the highly up-regulated genes showed a very low expression during normal condition. To name a few genes such as *LRRK168, 113.1*, and *135.3* that were extremely low expressing in normal stages, were highly up-regulated in various stresses. The quantitative RT PCR also showed similar expression profile of selected genes (Figure 6F). The *LRRK* genes *113.2, 180*, and *55.3* were down-regulated during each stress, while *26* and *177* were up-regulated in DS_1 h. The phylogenetic grouping showed that majority of these highly affected genes belongs to group VII, followed by IV and VI (Figure 6D).

Salt stress is another major abiotic stress affecting productivity of many crops. Herein, we analyzed the expression pattern of *TaLRRK* genes in root affected by salt stress using the earlier reported high-throughput RNA sequence data (Zhang et al., 2016). A total of 429 genes were affected by ≥2 folds up or down regulation (File S13), out of which top 50 were considered for detailed analysis. The heat map analysis showed that the highly affected *TaLRRK* genes could interestingly classified into two contrasting groups of continuously up and down regulated genes over an increase in exposure time of salt (Figure 6C). The RT PCR of selected genes also showed similar expression pattern (Figure 6G). The *LRRK* genes *26, 31.2, 55.3, 178*, and *180* were incrementally up-regulated with exposure time, while *LRRK177* was down-regulated. The *LRRK7, 67*, and *135.3* were down regulated in most of the treatments, while up-regulated in a few. However, *LRRK113.2* was up-reglutated in each treatment except salt_24 h. The phylogenetic classification indicated that most of these up and down regulated genes belonged to group VII and group III, respectively (Figure 6D). A few noticeably up regulated genes were *LRRK52, 55, 28*, and *153*, while down regulated genes were *LRRK198, 73, 205*, and *133*. Intriguingly, we observed that some of these highly up-regulated genes (*LRRK153.1, 51.1, 31.2*) were extremely low expressing under normal conditions, which indicated their specific role in salt stress response.

## Discussion

The LRRKs comprise a major subfamily of RLK. The *LRRK* genes play vital role in majority of biological activities of plants including growth and developments to stress management (Shiu and Bleecker, 2001; Shiu et al., 2004; Diévart and Clark, 2003). However, their functional characterization has still not been performed in *T. aestivum*, the most important crop plant of the world. This could be due to several factors like complex allohexaploid (2n = 6x = 42; AABBDD) genome, unavailability of genome sequence data and others. The availability of chromosome based genome information, and various developmental stage related and stress responsive RNA seq data in recent years facilitated the genome wide characterization of various gene families in *T. aestivum* (IWGSC, 2014; Zhang et al., 2014, 2016; Liu et al., 2015; Pingault et al., 2015; Shumayla et al., 2016). Since the LRRKs are responsible for various essential functions in plants (Shiu et al., 2004), here we have carried out a comprehensive analysis of these genes in *T. aestivum*.

Blast search of known protein sequences from model plants like arabidopsis, HMM blast and pfam blast are the standard procedures for the identification of new gene family sequences from non-model plants. Similar procedure was followed to characterize *LRRK* genes from various plants earlier (Zan et al., 2013; Rameneni et al., 2015; Wei et al., 2015). Here, we identified 531 non-redundant *TaLRRK* genes following the similar procedure, which were further confirmed by blast search against NCBI-conserved domain, PROSITE-Scan and SMART databases. Since the LRRK is the largest subfamily of RLK (Fischer et al., 2016), the number of *TaLRRK* genes identified was consistent with our expectation. About 213, 234, 303, 309, 382, and 467 *LRRK* genes are earlier reported in arabidopsis, tomato, rice, brassica, poplar, and soybean genomes, respectively (Shiu and Bleecker, 2001; Shiu et al., 2004; Sun and Wang, 2011; Zan et al., 2013; Rameneni et al., 2015; Wei et al., 2015; Zhou et al., 2016). The number of *TaLRRK* genes was fairly higher than the earlier studied plants, which might be due to the large hexaploid genome of *T. aestivum* (IWGSC, 2014). Similarly the higher number (467) of *LRRK* genes are reported from soybean, another polyploid crop plant (Schmutz et al., 2010; Zhou et al., 2016).

The allohexaploid genome of *T. aestivum* was originated as a result of hybridization of three (A, B, and D) diploid sub-genomes (Marcussen et al., 2014), which indicates the contribution of each sub-genome in composition of various gene families of *T. aestivum* (Shumayla et al., 2016; Zeng et al., 2016). Further, the contribution of number of genes from a sub-genome would be similar to their number in progenitor genome, unless they are not duplicated or deleted in due course of evolution after polyploidization. The genome wide distribution analysis indicated that the *TaLRRK* genes were also derived from individual sub-genomes (Figure 1A), while their number on A and D sub-genomes was quite lower than the number of *LRRK* genes predicted in *T. urartu* and *Aegilops tauschii*, which are progenitors of these sub-genomes, respectively (Marcussen et al., 2014). This indicated deletion of certain *TaLRRK* genes from these sub-genomes during evolution of *T. aestivum* after hybridization. The *TaLRRK* genes were also found to be distributed on each chromosome but at diverse frequency, as earlier reported in various other plants (Rameneni et al., 2015; Wei et al., 2015). For example, maximum and minimum number of *TaLRRK* genes was located on chromosome 2B and chromosome 5A, respectively. Similarly, chromosome 5 and chromosome 4 in arabidopsis, and chromosome 1 and chromosome 10 in brassica contain the highest and lowest number of *LRRK* genes, respectively (Rameneni et al., 2015; Wu et al., 2016).

The probability of occurrence of homologous genes has increased thrice in *T. aestivum* due to its allohexaploid genome (AABBDD). Hypothetically, each *T. aestivum* gene should have a minimum of three homologous genes, at least one from each sub-genome. These may also called as homeologous genes because of their localization on homeologous chromosomes. Here, we observed 531 *TaLRRK* genes in *T. aestivum*, which could be grouped into 233 distinct clusters of one or more homologous genes. The total number of *TaLRRK* genes was not actually thrice to their number in progenitor genomes like *T. urartu* and *Ae. tauschii*. Further, we could not detect three homeologous genes in case of each cluster. The majority of *TaLRRK* genes were single without any homeologous/homologous sequences, while a few had large number (up to 20) of homologous genes. This might be due to the consequences of deletion and/or duplication of certain *TaLRRK* genes during the process of evolution and acclimatization of *T. aestivum*. Similar kinds of deletion and duplication events are also reported in other gene families *T. aestivum* (Shumayla et al., 2016; Zeng et al., 2016). Moreover, the number of distinct *TaLRRK* genes clusters was comparable to the number of *LRRK* genes reported\predicted in various other diploid plants like arabidopsis, rice, *T. urartu, Ae. tauschii* and others (Shiu et al., 2004; Sun and Wang, 2011; Rameneni et al., 2015; Wei et al., 2015).

Genome hybridization, duplications, and inter-chromosomal rearrangements of various genes are reported as major events during the evolution of *T. aestivum* genome (Choulet et al., 2010; Marcussen et al., 2014; Akpinar et al., 2015; Glover et al., 2015). The role of paralogous genes evolved by duplication events (DE) are also reported in the evolution of lectin receptor kinases of *T. aestivum* (Shumayla et al., 2016), which indicated the role of duplication in the evolvement of other sub-families of RLK in this plant. Therefore, the frequency of various DEs was also analyzed in case of *TaLRRK* genes, following the established method (Shumayla et al., 2016). A large number of DEs were predicted in case of *TaLRRK* genes, which were distributed on various sub-genomes and chromosomes. About 25% of DEs were classified as TD and SD, while the majority of them could not further categorized due to the localization of one of both paralogous gene(s) on scaffolds. This is probably due to the unavailability of complete genomic data of *T. aestivum*. The occurrence of repeat sequences and transposable elements in the genome positively favors the gene duplication during evolution (Magadum et al., 2013). The *T. aestivum* genome is highly rich in these elements (Choulet et al., 2010, 2014; Marcussen et al., 2014; Glover et al., 2015), which might encourage the DEs in various gene families including *TaLRRKs*. The role of DEs in the expansion of LRRK gene family is earlier reported in various other plants like poplar, tomato, brassica and arabidopsis (Shiu and Bleecker, 2001; Shiu et al., 2004; Sun and Wang, 2011; Zan et al., 2013; Rameneni et al., 2015; Wei et al., 2015). These results along with earlier reports from other plants indicated that the gene duplication played critical role in diversification and neofunctionalization of *LRRK* genes during evolution.

The orthologous genes of *TaLRRKs* in rice and arabidopsis were identified by bidirectional best hit approach. Since both monocots and eudicots are evolved from common ancestor (McClung, 2010), the evolutionary relationship in the form of orthologous genes is obvious between them. Such kind of relationship has been reported in various gene families and plants in earlier studies (Sun and Wang, 2011; Rameneni et al., 2015; Wei et al., 2015; Shumayla et al., 2016).

The functional annotation of *TaLRRK* genes by blast search against various databases like NCBI-nr, UNIPROT and UNIREF further validated their identity. The *TaLRRK* sequences showed homology with most of the functionally characterized LRRKs like BRI1, ERL, SERK, CLAVATA from various plants including model plant arabidopsis. These LRRK proteins are earlier reported to be involved in various developmental and stress related activities (Wu et al., 2016). The GO mapping provides information about the characteristic features of various genes across the taxa. Since the LRRKs are membrane proteins (Torii, 2004), the GO analysis indicated membrane bound nature of each TaLRRK protein. Further, the prediction of binding and catalytic properties in TaLRRK proteins supported ligand interacting and enzymatic nature of LRR and kinase domains, respectively (Diévart and Clark, 2004; Torii, 2004). The GO mapping also predicted TaLRRKs role in signaling, stimulus response, and various other metabolic processes, which depicted their diverse function in plant development and defense mechanism. Similar GO categorization is also reported for brassica LRRKs (Rameneni et al., 2015).

The gene structure analysis showed variation in the presence of number of introns in various *TaLRRK* genes. The number of introns in *TaLRRK* genes was varied from zero to a maximum of 27 introns, wherein the majority of genes were single intronic. The variation in the number of exon/intron composition in *LRRK* genes is reported earlier in various plants. The single intronic *LRRK* genes are also reported as abundant class in rice, tomato, and poplar. Further, a maximum of 26 introns were reported in poplar, rice and tomato, while 24 in brassica (Sun and Wang, 2011; Zan et al., 2013; Rameneni et al., 2015; Wei et al., 2015). Similar complexity in *ERECTA* gene structure was reported in arabidopsis (Karve et al., 2011), which was predicted as an ortholog of *LRRK69.1* gene of *T. aestivum*. Previous studies suggested that introns influence the expression of genes, which has been experimentally validated with *ERECTA* gene in arabidopsis (Morello and Breviario, 2008; Karve et al., 2011; Chorev and Carmel, 2012). The significant expression of a few high intronic *TaLRRK* genes was also observed in certain tissues; however we could not establish a correlation between number of introns and expression pattern.

The length, molecular weight, and pI of TaLRRK proteins also varied as reported in various other plants (Sun and Wang, 2011; Zan et al., 2013; Rameneni et al., 2015; Wei et al., 2015). Since the majority of RLK are membrane protein and associated with the plasma membrane (Torii, 2004). Therefore, presence of a transmembrane region and signal peptide is the signature of the membrane RLK (Shiu and Bleecker, 2001). Occurrence of one or more transmembrane regions and a signal peptide indicated membrane bound nature of most of the TaLRRK proteins and majority of them were localized on plasma membrane. This indicated similar nature of TaLRRK proteins as reported in other plant species (Zan et al., 2013; Rameneni et al., 2015; Wei et al., 2015).

Analysis of domain composition showed the occurrence of 12 different types of domains in TaLRRK proteins, which were found in various combinations, where as kinase domain was consistently present at the C-terminus of each protein (Figure 3B). These diverse domain compositions also indicated functional divergence in various TaLRRK proteins. The diversity in domain composition has also been reported in other plant species like rice and brassica (Sun and Wang, 2011; Rameneni et al., 2015). Mostly the variation in domain composition is found at N-terminus and especially in extra-cellular domain of LRRKs, which supported the deviation in types of signal perception by LRRK proteins. This might be a possible reason for their diverse biological role in plant life cycle. Most of the TaLRRK proteins consisted of numerous types of LRR domains. Though the specific function of each type of LRR domain is not known, but largely they are tandemly repeated leucine rich motifs involved in protein-protein interactions (Bella et al., 2008). Malectin and P-loop NTPase domains were also found in a few TaLRRK proteins, which are probably involved in interaction with glycan and nucleic acid moieties (Leipe et al., 2004; Schallus et al., 2008), however their exact role in LRRK proteins is not established. Similarly a few TaLRRKs consisted of Aldolase II and Herpes_gE domains with unknown function. Several conserved motifs were also predicted in both extracellular LRR and intracellular kinase domains (Figure 3C; Table S2). Most of these motifs are also present in various other plant species (Zan et al., 2013; Rameneni et al., 2015; Wei et al., 2015). The organization of conserved amino acids (GxIPxxLxxLxxLxxLxLxxN) in LRR motif was also found to be highly conserved in both monocot and dicot plants like rice, tomato, and others (Sun and Wang, 2011; Rameneni et al., 2015; Wei et al., 2015). A few conserved motifs were found near the important sites like ATP binding sites, substrate binding sites, and activation loop of kinase domain, which play critical role during signal transduction (Clouse, 2011).

The phylogenetic relationship was analyzed using full-length TaLRRK protein sequences, because it provided excellent evolutionary inference. Full length sequences were used in case of LRRK phylogenetic analyses of other plant species (Sun and Wang, 2011; Rameneni et al., 2015; Wei et al., 2015). The identified homeologous and/or homologous sequences were closely grouped, which further established high sequence homology and evolutionary relation between them. The predicted paralogous sequences were also clustered together, which indicated their similar origin. Similar domain architecture and LRR motifs organization in most of the clades indicated functional correlation between these proteins, which has been observed during expression analysis and discussed below. Further, comparable organization of exon/intron pattern in various sub-groups and clades indicated a strong evolutionary association between them at gene level also. Similar type of gene structure and evolutionary relationship between LRRKs are also reported in other plant species like tomato, rice brassica and poplar (Sun and Wang, 2011; Zan et al., 2013; Rameneni et al., 2015; Wei et al., 2015).

The expression of a gene in a particular organ can be used as a source of information for identifying its function in that organ. Thus, the expression data of 531 *TaLRRK* genes was extracted from the available whole transcriptome dataset of *T. aestivum* from five different organs (root, stem, leaf, grain, and spike), each with three developmental stages (Pingault et al., 2015). The genes with at least 10RPKM expression values in one or more developmental stages were used for detailed analysis. It might be possible that these genes played critical roles in development of various organs, while the remaining genes might be involved in other functions like stress management. The organ specific expression indicated divergent role of various *TaLRRK* genes. Most of the genes were specifically expressed in particular organ like leaves or roots or grains, which indicated their specific role in the development of that organ. The organ specific variation in expression of *LRRK* genes is also reported in arabidopsis, tomato, brassica, soybean, and poplar (Gou et al., 2010; Zan et al., 2013; Rameneni et al., 2015; Wei et al., 2015; Zhou et al., 2016). An evolutionary functional association between organ specific *TaLRRK* genes was also established as majority of high expressing genes from a particular organ were clustered into same phylogenetic group.

The developmental stage specific expression profiling in various organs indicated that the genes, which were earlier categorized as organ specific, were found to be developmental stage specific. Since the functions of various LRRK proteins of arabidopsis have been established (Wu et al., 2016), the putative functions of few TaLRRK proteins were predicted based on their sequence homology. The arabidopsis SERK2 reported to be involved in anther development, maturation and brassinosteroid signaling in roots, anthers, and seedlings (Colcombet et al., 2005; Roux et al., 2011; Wu et al., 2016), showed homology with LRRK94.3, which was also highly expressed in root, grain and spike. Similarly, various other proteins of arabidopsis showed sequence homology with TaLRRK proteins such as BAM1-3 (LRRK60, 122, and 177) and CLAVATA (LRRK41) that are involved in shoot and floral growth, and cell differentiation, GSO (LRRK101) and RPK1 (LRRK116.2) in embryo development, ERL1-2 (LRRK69.2) in organ growth and floral development, BRI1 (LRRK90.1 and 90.4) in shoot vascular development, HSL2 (LRRK107) in abscission of flower and FEI2 (LRRK192.1) in cell wall biosynthesis (Clark et al., 1997; Delgado et al., 2004; Shpak et al., 2004; Deyoung et al., 2006; Nodine et al., 2007; Stenvik et al., 2008; Tsuwamoto et al., 2008; Xu et al., 2008).

The expression pattern of *TaLRRK* genes was also studied using high-throughput transcriptome data generated after 24 h of infection of two common fungal pathogens Bgt and Pst, which are responsible for powdery mildew and stripe rust diseases, respectively (Zhang et al., 2014). We observed a differential response of *TaLRRK* genes for these two pathogens though both of them are fungal species. This indicated that a diverse mechanism of signal perception and transduction is involved in these two pathogen infections. Further, several highly affected genes during fungal infections were actually very low abundant in normal plant developmental stages, which established their specific role in these biotic stress responses. The differential expression patterns of *LRRK* genes under fungal attack have also been reported in other plants like poplar (Zan et al., 2013). Though none of the earlier studies have reported characterization of *LRRK* genes during Pst and Bgt infection, several arabidopsis LRRK proteins are reported to be involved in biotic stress management (Wu et al., 2016). LRRK proteins like PSKR1, BAK1, BRI1, SERK, and FLS of arabidopsis that are involved in various defense responses (Gomez and Boller, 2000; Nam and Li, 2002; Roux et al., 2011; Wang et al., 2011; Mosher et al., 2013; Wu et al., 2016), showed homology with LRRK126.1, 90.3, 188, 39.3, and 220 of *T. aestivum*, respectively. This indicated that these genes might have similar function as their arabidopsis homologs, which needs to be functionally characterized in future studies.

Plants are exposed to various abiotic stresses like heat, drought, salt and others, which drastically affect the yield by distressing various biochemical and physiological processes (Prasad et al., 2011; Pradhan et al., 2012; Izadi et al., 2014; Yang et al., 2014). Since the LRRKs are reported to be involved in abiotic stress responses (Torii, 2004), we analyzed the effect of various abiotic stresses on the expression of *TaLRRK* genes using high throughput transcriptome data (Liu et al., 2015; Zhang et al., 2016). Since heat and drought generally act in synergistic manner (Liu et al., 2015), the expression of *TaLRRK* genes was analyzed under heat, drought, and their combined stresses as well. Several *TaLRRK* genes were found to be differentially expressed, which indicated their role in these stress conditions. Diverse response of *TaLRRK* genes during DS and HS indicated variation in their modus operandi. However, a similar trend of up or down regulation was observed for most of the affected genes during HS and DS. In case of HD, majority of genes showed assorted expression, which revealed cross talk between signaling pathway during these stresses. Majority of up-regulated *TaLRRK* genes were very low expression during normal condition, which indicated their role in these stresses. The phylogenetic analysis revealed that most of the highly affected genes were clustered into group VII (Figure 6D). It is noteworthy that the group VII consisted of least expressing genes during normal stages of various organ development (Figures 5C-G). The results indicated that these group VII genes were not only associated in their expression pattern, but also evolutionarily linked with each other. The role of *LRRK* genes in abiotic stress management is reported in various plants like rice and potato (Diévart and Clark, 2004; Park et al., 2014; Wu et al., 2016). The OsGIRL1 of rice, which showed homology with LRRK29.1 of *T. aestivum*, was earlier reported to be involved in heat, salt, osmotic, and gamma radiation stresses (Park et al., 2014).

Salt stress adversely affects the productivity of several crops, and it has been earlier reported that the amount of land affected by salinity is increasing (Wang et al., 2003). Salt stress induces various complex biochemical, molecular, cellular, and physiological changes in plants (Wang et al., 2003; Tuteja, 2007; Munns and Tester, 2008). Salt induced changes in transcriptome expression pattern are earlier reported in the root of *T. aestivum* (Zhang et al., 2016). The *TaLRRK* genes were also found to be affected by salt stress. Interestingly, the affected *TaLRRK* genes could classify into two groups with constant up and down regulation with the exposure time of salt. Most of these up and down regulated genes were clustered into phylogenetic group VII and III, respectively. The results indicated evolutionary association between the genes showing similar behavior during salt stress. The role of *LRRK* genes in salt stress management is also reported in rice and arabidopsis (Ten Hove et al., 2011; Park et al., 2014).

In conclusion, the present study was focused on the analyses of various characteristic features and expression patterns of *TaLRRK* genes of *T. aestivum*, a major food crop. Similar to the other plant species, *TaLRRK* was found to be one of the largest gene sub-family, which was categorized into eight distinct phylogenetic groups. The gene and protein structure, domain architecture, motifs arrangement, and composition were mostly conserved in various phylogenetic sub-groups. Occurrence of numerous paralogous genes indicated the role of duplication events in the expansion of this gene family during the evolution process. The expression profiling indicated the role of *TaLRRK* genes in development of various organs under normal and different stress conditions, however the specific role of individual gene needs to be validated by functional genomics tools in future studies. The analysis of major responsive genes in various stress conditions indicated specific roles of most of the *TaLRRK* genes. For instance, 33, 35, and 31 out of top 50 affected *TaLRRK* genes were specifically related to biotic, salt, and heat/drought stress, respectively (Figure 6E). Moreover, *LRRK134.1, 134.2*, and *134.3* genes were affected during all stress conditions. These results may provide valuable information to investigate the function of individual *TaLRRK* genes in plant development and stress responses using modern genome editing and functional genomics tools.

## Author contributions

SU conceived the idea and designed the experiments. S, SS, and RK performed the experiments. S, VM, KS, and SU analyzed the data. S and SU wrote the manuscript. VM and SU finalized the manuscript.

### Conflict of interest statement

The authors declare that the research was conducted in the absence of any commercial or financial relationships that could be construed as a potential conflict of interest.

## Supplementary material

File S1 **Distribution of 531 LRRKs on *T.aestivum* A, B, and D sub-genomes and chromosomes**.

File S2 **Predicted number of LRRKs in *T.urartu* and *A.tauschii***.

File S3 **Unigene clustering, nomenclature, chromosomal localization, and characterization of LRR receptor like kinases**.

File S4 **Genome and chromosome wide distribution of Duplication events (paralogs) in LRR receptor like kinases**.

File S5 **Predicted orthologs of LRR receptor like kinases in *Oryzae sativa* and *Arabidopsis thaliana***.

File S6 **Annotation and gene ontology mapping of LRR receptor like kinases using B2G tool at *e*-value 10-6**.

File S7 **Annotation of LRR receptor like kinases using various databases at *e*-value 10-6**.

File S8 **Analysis of various domain composition of LRR receptor like kinases**.

File S9 **Analysis of domain composition and LRR repeat of LRR receptor like kinases in various phylogenetic groups**.

File S10 **Expression analysis of LRR receptor like kinases in various organs and at each developmental stage**. Only genes with 10 rpkm value are listed.

File S11 **Expression analysis of LRR receptor like kinases under biotic stress**. The genes with 2 fold up or down regulation are shown in pink and green color cells respectively.

File S12 **Expression analysis of LRR receptor like kinases under heat or drought stress**. The genes with 2 fold up or down regulation are shown in pink and green color cells respectively.

File S13 **Expression analysis of LRR receptor like kinases during salt stress**. The genes with 2 fold up or down regulation are shown in pink and green color cells respectively.

Table S1 **List of primers used for quantitative real time PCR**.

Table S2 **Conserved motifs predicted in TaLRRK protein sequences and their corresponding motifs in other plants**.

Table S3 **Characterization of TaLRRK proteins in various phylogenetic groups**.

## Abbreviations

AA: Amino acids
DS: Drought stress
F: folds
h: hour
HD: Combined heat and drought stress
HS: Heat stress
SD: segmental duplication
TaLRRK: *T. aestivum* Leucine rich repeat receptor like kinases
TD: Tandem duplication.
