# Supplementary material for: Genomic Dissection and Expression Profiling Revealed Functional Divergence in Triticum aestivum Leucine Rich Repeat Receptor Like Kinases (TaLRRKs)
Source: Front Plant Sci. 2016 Sep 22;7:1374. doi: 10.3389/fpls.2016.01374 (PMC5031697; doi:10.3389/fpls.2016.01374)
Supplement: Table S2 — Conserved motifs predicted in TaLRRK protein sequences and their corresponding motifs in other plants. [file Table2.DOC]

**Table S2.** Conserved motifs predicted in TaLRRK protein sequences and their corresponding motifs in other plants.

| **S. No.** | **Sequence** | **Corresponding motifs in other plants** | | | |
| --- | --- | --- | --- | --- | --- |
|  |  | **Brassica**  (Rameneni et al., 2015) | **Poplar**  (Zan et al.,  2013) | **Rice**  (Sun et al.,  2011) | **Tomato**  (Wei et al.,  2015) |
| M1 | **E**[Y/L][g/a]x[t/g][S/T]xxxx[v/a][S/T]xK[G/S]**DVYSFG**[V/I][V/L]Lx**LE**[L/M][L/I]**TG**[K/R]x**P**xdxxxxd[n/y][g/p] | EKSDVYSFG[V/I][V/L]LLE[L/I][L/I/V]TG[K/R][R/K][P/A] | l/l**G**x**G**GfGxV**Y**K/rA/GxL/mx | xxNlI/L**G**x**G**gf**G**x**VY**KG/axLxxG | xNl[I/L]**G**x**G**gf**G**x**VY[K**/r]G/a |
| M2 | [T/S]x[T/R][v/l][a/r]**G**TX**GV**[i/l][A/P]**P** |  | xA**GT**/sx**GY**x**APEY**axT | x**GT**/si**GY**i**APEY**g/axx | T/sxva**G**T/sx**GY**i**APEY**a/gxtgkvT/s |
| M3 | **H**[R/C]**D**[l/i]**K**[S/P]**SNILLD**Gx[d/n][m/f]xA[k/h][V/I][s/a]**D**x**FGL**[A/S][R/K] |  | x**R**lk/n**I**/vAxG/dV/a**A**xG/A**L**xY**LH** | LdW/lxxRlxx**I**AlG/Dvv**A**xG/AxY**LH** | **W**xx**R**lk**I/**vAl**G/**dv/a**A**r**G/**a**L**x**YLH** |
| M4 | **G**n[D/E][f/e][k/r]x[a/l]**L**VYE[Y/F]M[p/e]N**G**[S/N]**L** |  | Ke**F**xx**E**v/ixx**L**gxi/lR**H**R**N**L/l**V**K**L** | xs**F**xx**E**c/vex**L**/isxv/iR**H**R**NL**/iVxL/  ixG/txCxxxd | xxkL**LV**YE/d**Y/**fMpN**G**S/t**L** |
| M5 | [I/V]**G**R**H**R**NL**V[k/p]L[i/l]G[Y/F]**C** | AE[V/I]Ex[L/I][GS][R/K][I/L/V]RH[R/P]NLVKL | **LVY**EY/FM/YxN**GSL**xxx**L** | **LVYE**Y/fMpN**G**S**L**xxx**L**Hxx | x**F**xx**E**v/iexL/ig/sxi/lR**H**r**N**L/i**V**/lx**L** |
| M6 | N[i/l][I/L]**G**[s/r]**G**[G/S][F/Y]**G**[s/t]**V**K[G/A]x**L** | [S/E]AN[V/I][L/I]GKGGFGTVY[K/R][G/A]VL | l/l**G**x**G**GfGxV**Y**K/rA/GxL/mx | xxNlI/L**G**x**G**gf**G**x**VY**KG/axLxxG | xNlI/L**G**x**G**gf**G**x**VYK**/rG/a |
| M7 | [F/L]xxG**N**[p/k][g/k]**LC**GxxxxA | FxGNPGLCGxPLxxC |  |  |  |
| M8 | L[N/D][L/V]**S**[F/Y]**N**[n/d]xL[n/s][e/s]**G**[p/e][V/I]x**P**x | LDLSNNxL[T/S]GPIPxE |  |  |  |
| M9 | [n/q]L[S/T]**G**[s/p]**IP**xx[S/F]L | xNxL[T/S]GE[I/L]Px[S/E][L/I/F]GNLTSLxxLDLSxNxL[T/S]GEIPxE[L/I] |  |  |  |
| M10 | [t/s][n/s]**L**[q/e]x**L**[D/N]**L**S[h/n]**N** | NLx[N/S]LQxLDLSNNx[F/L] |  |  |  |
| M11 | **L**[l/q]xx**L**[D/N]L**S**x**N**[n/s]**L**[S/T]**G**xI**P**[q/d][s/e]L[G/S]nL | xxL[D/N]LSYNN[L/F][S/T]GxIP |  | l/f/iGx**L**xx**L**xx**LDLS**x**N**xLt/s**G**xI**P**x |  |
| M12 | [S/G][r/c]**N**x[L/F][s/t]**G**[s/t]**IP** | GNLTSLxVL[D/N]LSNNN[L/F][S/T]G[S/E]IP |  |  |  |
| M13 | [G/P][l/v]E[N/L][G/S][N/Q]**Y**CxxL[e/q][s/v]L[d/n]**L**[s/d][N/G]**P** |  |  |  |  |
| M14 | **F**L[T/S]**L**[T/A]NC[T/S][Q/N]**L**[t/q]xLxL[S/G]xN[n/s][L/F]x**G**xL**PP** |  |  |  |  |
| M15 | **LQ**[E/H][l/v][I/V]D[L/F]S[A/P][a/g][k/s]**N**x[l/y][S/T]**G**[p/s][I/W]x**P**x[n/s][L/F][S/G]xL | [G/S]NLxSLEYL[N/D]LSxNN[F/L]SGS[I/V/L]P |  |  |  |
