# Supplementary material for: Genomic Dissection and Expression Profiling Revealed Functional Divergence in Triticum aestivum Leucine Rich Repeat Receptor Like Kinases (TaLRRKs)
Source: Front Plant Sci. 2016 Sep 22;7:1374. doi: 10.3389/fpls.2016.01374 (PMC5031697; doi:10.3389/fpls.2016.01374)
Supplement: Table S3 — Characterization of TaLRRK proteins in various phylogenetic groups. [file Table3.DOC]

**Table S3.** Characterization of TaLRRK proteins in various phylogenetic groups.

| **Phylogenetic groups** | **No. of Genes** | **Molecular weight (kDa)** | | **pI** | | **Protein length (AA)** | | **LRR repeat** | | | **No. of Introns** | | | **Signal peptide** | **Arabidopsis**  **Homologs** |
| --- | --- | --- | --- | --- | --- | --- | --- | --- | --- | --- | --- | --- | --- | --- | --- |
|  |  | Average | Range | Average | Range | Average | Range | Average | | Range | | Average | Range |  |  |
| Group  I | 2 | 123.05 | 115-131 | 6.94 | 6-8 | 1092.5 | 1020-1165 | 7.5 | 7-8 | | | 6.5 | 5-8 | 0% |  |
| Group  II | 42 | 69.13 | 43-100 | 6.2 | 5.3-9.2 | 674.7 | 413-932 | 5.2 | 1-9 | | | 11.2 | 0-18 | 50% | MRH1,  SERK2 |
| Group III | 128 | 73.7 | 36-103 | 6.3 | 5.29-9.67 | 711 | 335-1015 | 5.1 | 1-22 | | | 11 | 0-23 | 46% | RPK2, BRI1, PSKR2,  SERK1, ISO1, EFR,  SERK2, TMK-1 |
| Group IV | 42 | 73.83 | 475-114 | 7.3 | 5.5-9.9 | 719 | 442-1068 | 8.28 | 1-19 | | | 1.4 | 0-3 | 72% | TMKL1, IMK3, IMR2 |
| Group  V | 69 | 89.5 | 65-80 | 6 | 5.4-9.6 | 898 | 251-1315 | 14.8 | 1-31 | | | 1.4 | 0-13 | 57% | RPK2, PSKR2, BRI1, FEI |
| Group VI | 86 | 82.6 | 39-133 | 5.6 | 4.8-9.2 | 855 | 366-1265 | 15.8 | 2-29 | | | 3.3 | 0-27 | 40% | GSO1,CLV1, ERL1, EFR, RLK7, BAM1, HSL1 |
| Group VII | 157 | 84.3 | 17.9-127 | 6.3 | 5.3-9 | 833 | 169-1181 | 16.7 | Jan-44 | | | 1.2 | 0-5 | 38% | EFR, FLS2 |
| Group VIII | 5 | 61.7 | 33-115 | 4.9 | 05-Jul | 721.2 | 309-1069 | 17.6 | 7-31 | | | 0.8 | 0-1 | 60% | EFR |
|  |  |  |  |  |  |  |  |  |  |  |  |  |  |  |  |
